# H11/HSPB8 Restricts HIV-2 Vpx to Restore the Anti-Viral Activity of SAMHD1

**DOI:** 10.3389/fmicb.2016.00883

**Published:** 2016-06-13

**Authors:** Ayumi Kudoh, Kei Miyakawa, Satoko Matsunaga, Yuki Matsushima, Isao Kosugi, Hirokazu Kimura, Satoshi Hayakawa, Tatsuya Sawasaki, Akihide Ryo

**Affiliations:** ^1^Department of Microbiology, School of Medicine, Yokohama City UniversityYokohama, Japan; ^2^Kawasaki City Health and Safety Research CenterKanagawa, Japan; ^3^Department of Regenerative and Infectious Pathology, Hamamatsu University School of MedicineHamamatsu, Japan; ^4^Infectious Disease Surveillance Center, National Institute of Infectious DiseasesTokyo, Japan; ^5^Division of Microbiology, Department of Pathology and Microbiology, Nihon University School of MedicineTokyo, Japan; ^6^Proteo-Science Center, Ehime UniversityMatsuyama, Japan

**Keywords:** virus–host interaction, wheat germ cell-free protein synthesis, AlphaScreen, Vpx, H11, HIV-2

## Abstract

Virus–host interactions play vital roles in viral replication and virus-induced pathogenesis. Viruses rely entirely upon host cells to reproduce progeny viruses; however, host factors positively or negatively regulate virus replication by interacting with viral proteins. The elucidation of virus–host protein interaction not only provides a better understanding of the molecular mechanisms by which host cells combat viral infections, but also facilitates the development of new anti-viral therapeutics. Identification of relevant host factors requires techniques that enable comprehensive characterization of virus–host protein interactions. In this study, we developed a proteomic approach to systematically identify human protein kinases that interact potently with viral proteins. For this purpose, we synthesized 412 full-length human protein kinases using the wheat germ cell-free protein synthesis system, and screened them for their association with a virus protein using the amplified luminescent proximity homogenous assay (AlphaScreen). Using this system, we attempted to discover a robust anti-viral host restriction mechanism targeting virus protein X (Vpx) of HIV-2. The screen identified H11/HSPB8 as a Vpx-binding protein that negatively regulates the stability and function of Vpx. Indeed, overexpression of H11/HSPB8 promoted the degradation of Vpx via the ubiquitin–proteasome pathway and inhibited its interaction with SAMHD1, a host restriction factor responsible for blocking replication of HIV. Conversely, targeted knockdown of H11/HSPB8 in human trophoblast cells, which ordinarily express high levels of this protein, restored the expression and function of Vpx, making the cells highly susceptible to viral replication. These results demonstrate that our proteomic approach represents a powerful tool for revealing virus–host interaction not yet identified by conventional methods. Furthermore, we showed that H11/HSPB8 could be a potential host regulatory factor that may prevent placental infection of HIV-2 during pregnancy.

## Introduction

To replicate and propagate, viruses utilize sophisticated mechanisms to hijack the machinery and materials of their host cells. In a virally infected cell, host proteins play crucial roles in multiple biological processes that promote viral replication by providing molecular architecture or functional assistance. To this end, viral proteins need to interact directly or indirectly with host cell proteins that are relevant to the viral life cycle. On the other hand, some host proteins operate as anti-viral factors to counteract or restrict viral replication within infected cells. In turn, certain viral proteins counteract these anti-viral proteins, resulting in sustained viral propagation. Therefore, a comprehensive understanding of dynamic host–virus protein interaction would greatly improve our understanding of the viral life cycle and pathogenesis.

Recent technological advances in high-throughput and quantitative proteomics have enabled us to comprehensively analyze biologically relevant protein–protein interactions. In particular, the development of cell-free protein synthesis (CFPS) systems has enabled high-yield production of active and functional proteins that can be used for biological analysis (Sawasaki et al., 2002; Endo and Sawasaki, 2006; Zawada et al., 2011). Moreover, advances in label-free protein detection techniques enable reliable, high-throughput analysis of protein–protein interactions (Sawasaki et al., 2007). Protein–protein interactions can be also predicted by approaches that combine bioinformatics and structural biology (Roy et al., 2010; Roche et al., 2015). Together, these techniques have contributed to the establishment of comprehensive protein interaction networks, which facilitate understanding of biological mechanisms, both in cells and in the context of viral infection.

Recombinant protein production and purification are essential steps for biochemical and functional characterization of virus and host proteins. In general, however, viral protein expression is limited due to the toxicity and insolubility of viral proteins in living cells (Harbers, 2014; Gagoski et al., 2016). Compared with conventional cell-mediated protein expression methods, the wheat germ CFPS system is more likely to produce properly folded, soluble, and functional virus-encoded proteins. Previous reports showed that the wheat germ system has several advantages over protein expression in other CFPS system such as *E. coli* or HeLa cells, including improved protein solubility and expression of toxic proteins such as viral antigens (Gagoski et al., 2016). Thus, the wheat germ CFPS system represents a rapid and high-throughput methodology for translation of genetic information into protein-mediated biochemical activities for use in virological research (Sawasaki et al., 2007).

Methods for detecting protein–protein interactions can be categorized into several types: most broadly, *in vitro, in vivo*, and *in silico* methods. Among *in vitro* methods, the AlphaScreen (derived from “Amplified Luminescent Proximity Homogeneous Assay”) technology offers a rapid and simple means for quantifying target protein–protein interactions using a non-radioactive bead-based detection method. Upon excitation at 680 nm, the donor beads, which contain the photosensitizer phthalocyanin, convert molecular oxygen to excited singlet oxygen with a 4 μs half-life. The singlet oxygen can diffuse up to 200 nm to make contact with a thioxene derivative on the AlphaScreen acceptor beads, resulting in amplified chemiluminescent emission between 520 and 620 nm. One donor bead can generate 60,000 singlet oxygens, resulting in exceptionally high signal amplification and permitting adaptation of the AlphaScreen assay to multi-well plate formats (Taouji et al., 2009). Thus, the AlphaScreen technology is suitable for high-throughput analysis of protein–protein interactions.

Viral proteins are controlled by post-translational modifications such as phosphorylation during infection (Nandi and Banerjee, 1995; Rajendra Kumar et al., 2005; Hemonnot et al., 2006; Kudoh et al., 2014). Phosphorylation acts as a molecular switch of target protein, thereby modulating their functions. We previously showed that HIV-1 Gag was regulated by the aPKC-mediated phosphorylation by using a human protein kinase library (Kudoh et al., 2014). Identification of human protein kinases that interact with viral protein could be effective approach to reveal a novel viral–host interaction. HIV-2 encodes an accessory protein Vpx that degrades SAMHD1, a host restriction factor. Although previous reports suggested that HIV-2 Vpx is phosphorylated during infection (Nandi and Banerjee, 1995; Rajendra Kumar et al., 2005), it still remains uncertain if Vpx phosphorylation indeed affects to functions of Vpx toward SAMHD1 degradation. Thus, we decided to investigate molecular interaction between human protein kinases with HIV-2 Vpx protein.

In this study, we performed a high-throughput screen of interactions between viral and host proteins using the wheat germ CFPS system and AlphaScreen. As an illustrative example, we analyzed the functional interaction between HIV-2 Vpx and host protein kinases in order to elucidate the function of Vpx protein. Furthermore, we describe the results of a pilot study designed to test the experimental feasibility of our *in vitro* assay system, and discuss the optimal strategy for characterizing virus–host interactions.

## Materials and Methods

### Viral DNA Constructs and Plasmids

HIV-2 reporter virus vectors pGL-ANΔ*Env*-Luc and pGL-StΔ*Env*Δ*Vpx*-Luc were kindly provided by Dr. Akio Adachi (Tokushima University, Tokushima, Japan). Plasmids expressing FLAG-tagged Vpx were kindly provided by Dr. Akio Adachi (Tokushima University, Tokushima, Japan; Khamsri et al., 2006). Vpx deletion mutants and H11 substitution mutants were generated by PCR-based molecular cloning procedures using PrimeSTAR Max (Takara Bio Inc, Shiga, Japan).

### Antibodies

Anti-FLAG (M2), anti-SAMHD1, and anti-vinculin mouse monoclonal antibodies were obtained from Sigma (St. Louis, MO, USA). Anti-HA (3F10) rat monoclonal antibody was obtained from Roche (Mannheim, Germany). Anti-Hsp22 (H11) rabbit polyclonal antibody was from Abcam (Tokyo, Japan).

### Cells and Viruses

HEK293 and HEK293T cells were cultured in DMEM (Gibco-BRL, Rockville, MD, USA) supplemented with 10% (V/V) fetal bovine serum (FBS; Gibco-BRL). Human villous trophoblasts (HVT; ScienCell Research Laboratories, USA, HVT were isolated from human placental villi and cryopreserved at passage primary culture). Pavlov et al. (2003) were cultured in Trophoblast Medium (ScienCell Research Laboratories, Carlsbad, CA, USA). THP-1 cells were cultured in RPMI containing 10% FBS. THP-1 cells were differentiated overnight with 50 ng/ml of phorbol 12-myristate 13-acetate (PMA; Sigma–Aldrich). Vesicular stomatitis virus G glycoprotein (VSV-G)-pseudotyped viruses were produced in HEK293T cells co-transfected with reporter virus and VSV-G plasmids using the calcium-phosphate method. Culture supernatants were collected, and HIV-2 particle yields were quantitated by p27 antigen capture enzyme-linked immunosorbent assay (ELISA; ZeptoMetrix, Buffalo, NY, USA).

### *In vitro* Protein Production

A total of 412 cDNAs encoding human protein kinases were generated as described previously (Tadokoro et al., 2010). The protein production method was also described previously (Sawasaki et al., 2002, 2007; Takai et al., 2010). Briefly, DNA templates containing a biotin-ligating sequence (bls) were amplified by split-PCR using cDNAs and corresponding primers, and then used in a GenDecoder protein production system (Cell Free Science, Ehime, Japan). For synthesis of HIV-2 Vpx protein, Vpx genes derived from the pGL-AN proviral plasmid were generated by split-PCR and used as templates in the Wheat Germ Expression kit (Cell Free Science).

### AlphaScreen-Based Protein–Protein Interaction Assays

AlphaScreen assays were performed as described previously (Tadokoro et al., 2010). All recombinant proteins were synthesized using the wheat germ CFPS system, as described above. For each protein kinase, 1 μl of crude recombinant biotinylated construct from the human kinase library was incubated with 1 μl of crude FLAG-Vpx or FLAG-DHFR in 10 μl of kinase assay buffer (100 mM Tris-HCl [pH 8.0], 10 mM MgCl2, 0.1% Tween-20, 0.1% BSA) at 37°C for 1 h in one well of a 384-well OptiPlate (PerkinElmer, Foster City, CA, USA). Using the AlphaScreen IgG (protein A) detection kit (PerkinElmer), 15 μl of detection mixture containing 100 mM Tris-HCl [pH 8.0], 0.01% Tween-20, 1 mg/ml BSA, 5 μg/ml anti-FLAG antibody (GE Healthcare, Buckinghamshire, UK), 5 ng streptavidin-coated donor beads, and 5 ng anti-IgG (protein A) acceptor beads were added to each well, followed by incubation at 26°C for 1 h. AlphaScreen signals were detected on an EnVision device (PerkinElmer) using the AlphaScreen signal detection program.

### *In vitro* Kinase Assays

Biotinylated-DHFR, H11, SAMHD1, and FLAG-Vpx proteins were synthesized in the wheat germ CFPS system, as described above. The synthesized proteins were purified using streptavidin-conjugated magnet beads (Promega, Madison, WI, USA) or Flag M2 beads (Sigma–Aldrich). Purified FLAG-Vpx proteins were then incubated with each biotinylated protein in a 50 μl reaction mixture containing 20 mM Tris-HCl (pH 7.5), 1 mM EDTA, 1 mM dithiothreitol, 150 mM NaCl, 5 mM MgCl_2_, 0.05% Tween-20, 100 μM ATP, and 2 μCi [γ-32P]ATP. The reaction mixture was incubated for 1 h at 37°C, and the products were subjected to electrophoresis on 10% SDS polyacrylamide gels and detected on a BAS2500 scanner (Fujifilm, Tokyo, Japan).

### Western Blotting

Cells were harvested at the indicated time points, washed with phosphate-buffer saline (PBS), and treated with lysis buffer (0.02% sodium dodecyl sulfate [SDS], 0.5% Triton X-100, 300 mM NaCl, 20 mM Tris-HCl [pH 7.6], 1 mM EDTA, 1 mM dithiothreitol) for 20 min on ice. Multiple protease inhibitors, 200 μM sodium vanadate, and 20 mM sodium fluoride were then added to the buffer. The samples were centrifuged at 18,000 *g* for 10 min at 4°C, and the clarified cell extracts were assayed for protein concentration using DC^TM^ protein assay kit (Bio-Rad, Hercules, CA, USA). Equal amounts of proteins (20–50 μg) were resolved by SDS-PAGE on 10% gels (acrylamide, 29.2; bisacrylamide, 0.8) in running buffer (250 mM glycine, 25 mM Tris, 0.1% SDS). The separated proteins were transferred to polyvinylidene difluoride membrane. The membranes were washed with blotting buffer (TBS containing 0.1%-Tween 20), and then blocked for 1 h at room temperature in 10% non-fat powdered milk in blotting buffer. Primary antibodies were added at appropriate dilutions in 3% bovine serum albumin in blotting buffer and rocked overnight at 4°C. The membranes were then washed in blotting buffer and incubated for 1 h at room temperature with a horseradish peroxidase–conjugated secondary antibody. Target proteins were detected using an enhanced chemiluminescence detection system (GE Healthcare). Images were processed with FluorChem FC2 (Alpha Innotech Corp. Tokyo, Japan), acquired using a cooled charge-coupled device (CCD) camera, and assembled using Adobe Photoshop CS5 Extended.

### Immunoprecipitation

HEK293 cells were co-transfected with HA-H11 and FLAG-Vpx, and treated with 20 μM of MG132 for 4 h before harvest. Harvested cells were lysed in lysis buffer (50 mM Tris-HCl [pH 8.0], 150 mM NaCl, 1 mM EDTA, 1 mM DTT) containing complete protease inhibitor cocktail (Roche Molecular Biochemicals, Indianapolis, IN, USA) and PhosSTOP phosphatase inhibitor cocktail (Roche Molecular Biochemicals). Lysates were cleared by centrifugation at 12,000 × *g* for 15 min, followed by pull-down with anti-FLAG M2 affinity Gel (Sigma) or anti-HA affinity Gel (Sigma). Samples were separated by SDS-PAGE and analyzed by Western blotting.

### Phosphatase Treatment

HEK293 cells were co-transfected with plasmids encoding HA-H11 and harvested at 24 h after transfection. Harvested cells were suspended with lysis buffer (0.02% sodium dodecyl sulfate [SDS], 0.5% Triton X-100, 300 mM NaCl, 20 mM Tris-HCl [pH 7.6], 1 mM EDTA, 1 mM dithiothreitol and Complete [Roche, Basel, Switzerland]) for 20 min on ice. The lysate was then incubated in reaction buffer and calf intestinal alkaline phosphatase (CIAP; Takara Bio Inc., Shiga, Japan) for 2 h at 37°C. The reaction was stopped by the addition of 2 x sample buffer.

### Single-Cycle Virus Release Assays

For HA-H11 overexpression assays, PMA differentiated THP-1 cells were transfected with HA-H11 plasmids with Lipofectamin 3000 (Thermo Fisher Scientific, Waltham, MA USA), and 24 h after transfection cells were infected with VSV-G-pseudotyped HIV-2 at a multiplicity of infection (MOI) of 2, and cultured for 2 days. For small interfering RNA (siRNA) targeting *H11* transfected assays, HVT cells were transfected with H11 specific siRNA, GGAGUUGAUGGUGAAGACCAAAGAU, purchased from invivogen (invivogen, San Diego, CA, USA) with using RNAiMAX (Thermo Fisher Scientific, Waltham, MA, USA), and 24 h after transfection cells were infected with VSV-G-pseudotyped HIV-2 at a MOI of 2, and cultured for 2 days. Cell lysates were prepared using HBST buffer (10 mM HEPES [pH 7.4] 150 mM NaCl, 0.5% Triton X-100) containing protease inhibitor cocktail (Roche, Basel, Switzerland).

### Tissue Collection

A fallopian tube resected from a patient with ectopic tubal pregnancy was retrieved from the archives of Seirei Hamamatsu General Hospital, Shizuoka, Japan.

### Immunohistochemistry

Unstained sections were deparaffinized and rehydrated prior to antigen retrieval. Antigen retrieval was performed in 10 mmol/L citrate buffer (pH 6.0) in a microwave oven. Sections were incubated with anti-Hsp22 antibody for 30 min at room temperature. After washing in PBS, the sections were incubated with peroxidase-conjugated universal immune-enzyme polymer, anti-rabbit solution (Histofine Simple Stain MAX PO-R Nichirei Biosciences, Tokyo, Japan), and then visualized with 3,3-diaminobenzidine (Sigma–Aldrich) and counterstained with hematoxylin.

### Ethical Statement

Ethical approval was obtained from the Ethical Committee of Seirei Hamamatsu General Hospital, Shizuoka, Japan (September 22, 2010, #917). A patient provided written informed consent for the collection of samples and subsequent analysis.

### Statistical Analysis

Statistical analysis was performed using the Excel Tokei software series (Esumi, Tokyo, Japan). Data are presented as means ± SD. One-way analysis of variance (ANOVA) and Student’s *t*-test were used for comparisons of continuous variables. *P* < 0.05 was considered significantly different.

## Results

### Design and Development of a High-Throughput Protein–Protein Interaction Screen

**FIGURE 1:**
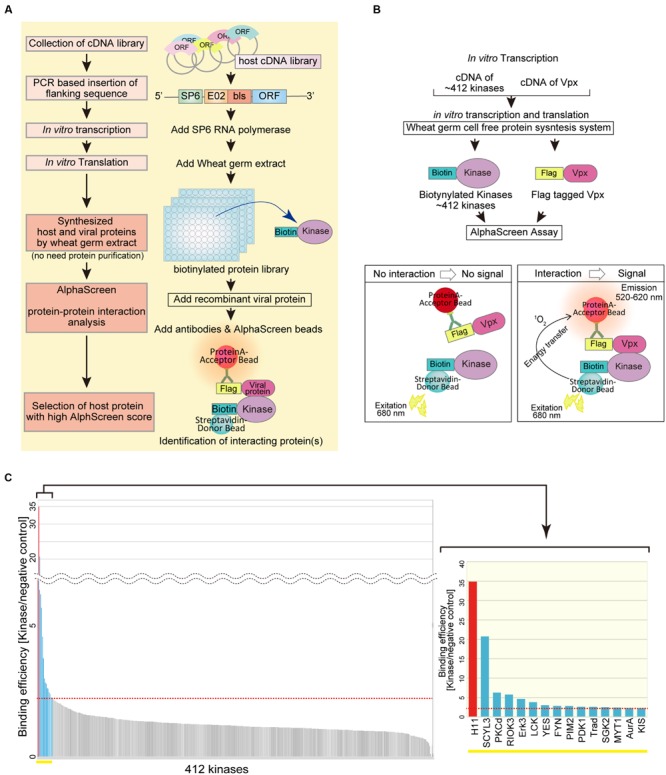
**AlphaScreen-based screening of virus–host proteins interactions. (A)** Schematic representation of the overall screening procedure. **(B)** Schematic representation of the AlphaScreen-based luminescent system used to screen for human protein kinases that interact with HIV-2 Vpx. All recombinant proteins were produced in the wheat germ CFPS system. Briefly, recombinant FLAG-tagged HIV-2 Vpx or dihydrofolate reductase (DHFR) was incubated with each protein kinase. Protein A–conjugated acceptor beads with anti-FLAG antibody and streptavidin-coated donor beads were added and bound to the tagged substrate. Upon laser excitation, the donor beads convert ambient oxygen to singlet oxygen. When a molecular interaction occurs between HIV-2 Vpx and a particular kinase, singlet oxygen transfers to the acceptor beads, activating emission of light at 520–620 nm. **(C)** HIV-2 Vpx–interacting kinases identified from a human protein kinase library by AlphaScreen. The binding efficiency of HIV-2 Vpx with each kinase was normalized relative to the luminescence of control DHFR.

To build a system for high-throughput protein–protein interaction screening, we utilized a wheat germ CFPS system to synthesize a set of proteins from a host cDNA library (**Figure 1A**). Linear DNAs used as translation templates for CFPS were PCR amplified from a plasmid library of human protein kinase cDNAs. In this step, the template DNAs were fused at the 5′ end to a specific sequence from the SP6 promoter, the E02 enhancer region, and a bls by split-primer PCR using appropriate primers (Sawasaki et al., 2008; Matsuoka et al., 2010). The templates were then transcribed by SP6 RNA polymerase, and the resultant mRNAs were translated in wheat germ extracts. The biotin ligation method yields a biotin label on the bls, allowing specific recognition of the target protein by Amplified Luminescent Proximity Homogenous Assay (AlphaScreen).

### Identification of Host Protein Kinases that Interact with HIV-2 Vpx

Protein kinases are enzymes that modify substrate proteins by chemical addition of phosphate groups. Phosphorylation usually results in structural and functional changes in the target protein. Therefore, we investigated functional modification of virus protein by host protein kinases. As an illustration, we sought to identify host protein kinases that functionally associate with the HIV-2 accessary protein Vpx. To this end, we quantitatively monitored protein–protein interactions using AlphaScreen (**Figure 1B**). The binding efficiency of HIV-2 Vpx with each kinase was normalized relative to the luminescent activity of DHFR protein, used as a negative control (**Figure 1C**). When a relative light unit per cutoff (RLU/Co) ratio of ≥2.25 was used as the threshold, we found 15 host kinases that could selectively interact with HIV-2 Vpx. Among them, the luminescence signal of H11 (also known as HSPB8) was significantly higher than that of other kinases. Our assay detected Fyn and Erk2 as Vpx interactor (S/N = 2.85 and 1.36, respectively), both of which have been already reported to phosphorylate Vpx (Rajendra Kumar et al., 2005; Singhal et al., 2006). Therefore, we focused on functional analysis of H11 as a previously uncharacterized Vpx-interacting factor.

**FIGURE 2:**
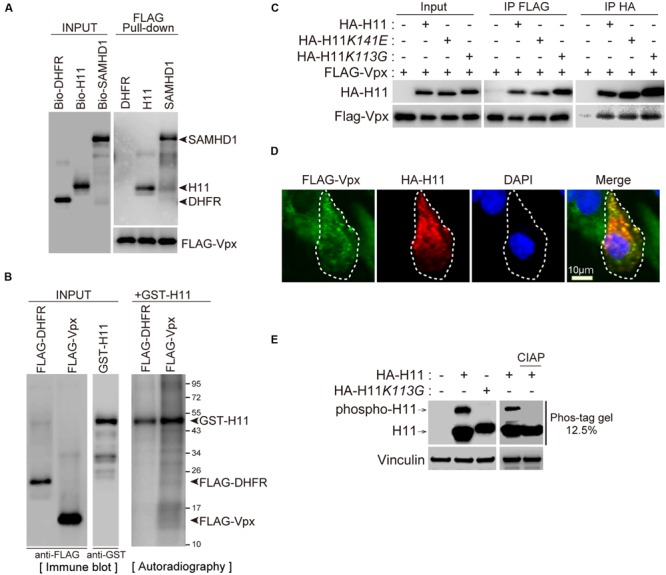
**H11 interacts with HIV-2 Vpx. (A)** Direct interaction between H11 and HIV-2 Vpx revealed by FLAG pull-down assays. Biotinylated H11, SAMHD1, DHFR, and FLAG-tagged HIV-2 Vpx were produced in the wheat germ CFPS system and affinity-purified. Both input and pull-down samples were subjected to western blotting with HRP-streptavidin and anti-FLAG antibody. **(B)** Immunoprecipitation of HIV-2 Vpx with H11. HEK293T cells were co-transfected with HA-tagged H11, H11*K141E*, or H11*K113G* and FLAG-tagged HIV-2 Vpx in the presence of MG132, and cell lysates were immunoprecipitated with anti-FLAG or anti-HA antibody 24 h later. Input and immunoprecipitation samples were then probed with anti-HA and anti-FLAG antibodies. **(C)** Confocal microscopy analysis of HA-tagged H11 and FLAG-tagged HIV-2 Vpx in HEK293T cells. Scale bar, 10 μm. **(D)** *In vitro* phosphorylation assay of H11. FLAG-tagged DHFR, Vpx, and GST-tagged H11 were produced in the wheat germ CFPS system and affinity-purified. H11-mediated phosphorylation was assessed by incubating recombinant FLAG-DHFR or FLAG-Vpx with recombinant active GST-H11 in the presence of [γ-^32^P] ATP. The reaction products were analyzed by autoradiography. Left panel shows a western blot of the input recombinant proteins. **(E)** H11 proteins mobility shifts in Phos-tag Gel. HEK293 cells were transfected with plasmids encoding HA-H11 or HA-H11*K113G* (100 ng). Right panel shows separation of HA-H11 with or without CIAP, phosphatase, treatment by Phos-tag affinity approach. The transfected cells were harvested at 24 h post-transfection, and then treated with CIAP. Equal amounts of cell lysates were subjected to a 50 μM phos-tag containing gel or a normal SDS-PAGE and then analyzed by Western blot analysis with HA or Vinculin antibodies. The arrow indicate the position of wild type H11. Phospho-H11 indicates phosphorylated form of H11.

To confirm the Vpx–H11 interaction, we performed pull-down assays. For this purpose, biotin-labeled H11, DHFR (negative control), SAMHD1 (positive control), and FLAG-tagged Vpx proteins were synthesized individually by wheat germ CFPS. Each biotin-labeled protein was mixed with FLAG-Vpx, and then immunoprecipitated with sepharose beads conjugated to anti-FLAG antibody (FLAG-beads). H11 was co-precipitated with FLAG-Vpx protein (**Figure 2A**). As reported previously (Hrecka et al., 2011; Laguette et al., 2011), SAMHD1 protein associated with FLAG-Vpx protein (**Figure 2A**). To further validate the interaction, we performed cell-based immunoprecipitation analysis using site-directed H11 mutants, a chaperon-like activity–deficient mutant K141E (Irobi et al., 2004; Shemetov et al., 2008; Shemetov and Gusev, 2011) and a kinase-activity–deficient mutant K113G (Smith et al., 2000). FLAG-Vpx was co-transfected with either HA- tagged wild type H11 or mutants in HEK293T, and cell lysates were immunoprecipitated using anti-FLAG or anti-HA affinity beads. The wild type and mutant forms of H11 were co-immunoprecipitated with Vpx, and Vpx protein was also co-precipitated with all three forms of H11 (**Figure 2B**), indicating that H11 interacts with HIV-2 Vpx *in vitro* and in cells, irrespective of its kinase or chaperon activity. Moreover, we found that HA- H11 was co-localized with FLAG-Vpx protein in the cytoplasm (**Figure 2C**).

We next investigated whether H11 could directly phosphorylate Vpx protein *in vitro*. Recombinant FLAG-DHFR or FLAG-Vpx proteins were synthesized and purified from wheat germ extract with FLAG beads and used as substrates for *in vitro* kinase assays. H11 did not phosphorylate Vpx, as was also the case for the negative control DHFR, although prominent auto-phosphorylation of H11 was observed (**Figure 2D**). We next analyzed the kinase activity of wild type and H11*K113G*, kinase activity deficient mutant, using Phos-tag affinity approach and western blot (Kinoshita et al., 2006). The phosphorylation of wild type H11 was detected as a distinct band shift. This was not a case with H11*K113G* mutants. The pre-treatment of cell lysates with calf intestine alkaline phosphatase (CIAP) resulted in the loss of band shift, indicating that shifted band represented phosphorylated H11 (**Figure 2E**).

**FIGURE 3:**
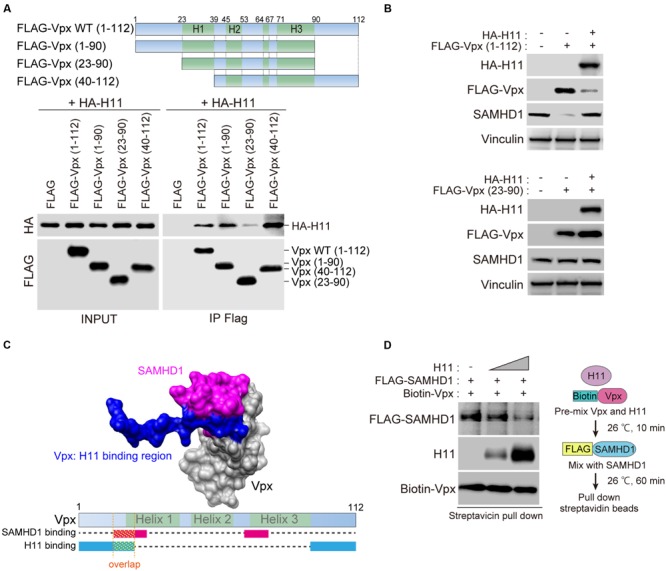
**Domains in HIV-2 Vpx that mediate physical interactions with H11. (A)** Top panel shows a schematic representation of the wild-type (WT; residues 1–112) and mutant Vpx proteins utilized in the FLAG-pull-down analysis. The name of each Vpx mutant is shown on the left. WT Vpx or the indicated mutants were co-transfected with HA-tagged H11 into HEK293T cells. The transfected cells were treated with 20 μM of MG132 for 4 h before harvest, and harvested at 48 h after transfection. Cell lysates were immunoprecipitated with anti-FLAG antibody. The immunoprecipitated proteins, as well as the starting material (INPUT), were subjected to SDS-PAGE and analyzed by western blot using anti-FLAG and anti-HA antibodies. **(B)** HEK293 cells were transfected with WT (top panel) or truncated mutant (bottom panel) FLAG-Vpx and HA-H11, as indicated. Whole cell extracts were prepared, and equal amounts of proteins from each sample were separated by SDS-PAGE and subjected to western blot analysis using the indicated antibodies. **(C)** Binding structure model of SAMHD1 C-terminal domain (magenta) and Vpx (gray) illustrated by Chimera (http://www.cgl.ucsf.edu/chimera/), based on the X-ray structure (PDB code: 4CC9). The H11-binding domain (blue) was overlaid on Vpx. Bottom panel shows schematic image of Vpx protein. The SAMHD1-binding region is indicated in magenta, and the H11-binding region is indicated in blue. **(D)** Recombinant biotinylated Vpx was pre-mixed with various amounts of H11 for 10 min before the addition of FLAG-tagged SAMHD1. After 60 min, the mixture was processed for pull-down with streptavidin-coated magnetic beads, as shown on the right. Bound proteins were detected by western blot (left panel).

Next, we attempted to identify the binding domain of Vpx with H11. To this end, we used the full-length form of Vpx (residues 1–112) and three deletion mutants (Vpx 1–90, 23–90, and 40–112) (**Figure 3A**). As shown in **Figure 3A**, full-length Vpx, C-terminally deleted Vpx (Vpx 1–90), and N-terminally deleted Vpx (Vpx 40–112) could precipitate with HA-H11 protein, but Vpx lacking both the N-terminal and C-terminal regions (Vpx 23–90) could not. These results demonstrated that either the N-terminus or C-terminus of Vpx is sufficient for the physical association with H11.

To better understand the functional relevance of the Vpx–H11 interaction, we investigated whether H11 could affect Vpx protein expression. Overexpression of HA-H11 repressed the expression of full-length Vpx but not the truncated mutant Vpx 23–90 (**Figure 3B**). Vpx can degrade SAMHD1 in a proteasome-dependent manner (Hrecka et al., 2011; Laguette et al., 2011). Consistent with this, the level of endogenous SAMHD1 decreased upon expression of wild-type (WT) FLAG-Vpx. The reduction in Vpx level resulting from HA-H11 overexpression restored endogenous SAMHD1 expression.

Previous reports showed that N-terminal regions of Vpx includes responsible domain for the interaction with SAMHD1 (Schwefel et al., 2014), and our results above indicate that H11 binds both the N- and C- termini of Vpx. Based on the crystal structure of the Vpx–SAMHD1 complex (Schwefel et al., 2014), the N-terminal SAMHD1-binding region within Vpx may overlap with the H11-binding region (**Figure 3C**), so that H11 may interfere with the SAMHD1-Vpx interaction. To test this hypothesis, we performed *in vitro* binding assays for the Vpx-SAMHD1 interaction in the presence of various amounts of H11. The results revealed that H11 inhibited the interaction between SAMHD1 and Vpx (**Figure 3D**). Collectively, our results suggest that H11 can not only enhance degradation of Vpx, but also directly inhibit its interaction with SAMHD1 for proteasomal degradation.

### H11 Degrades Vpx in a Proteasome-Dependent Manner

**FIGURE 4:**
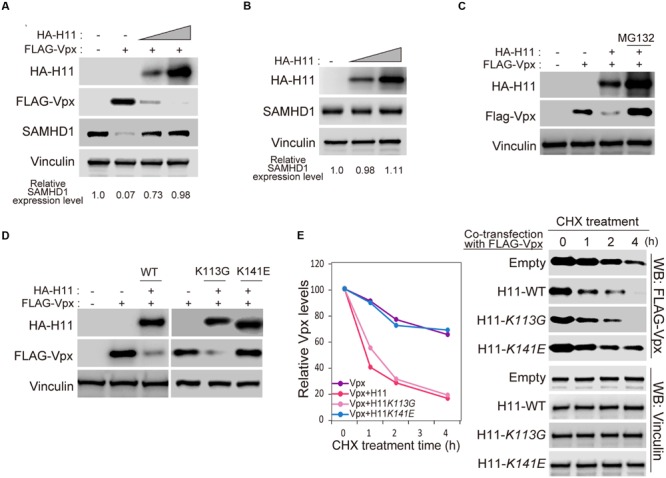
**Aberrant expressed H11 degrades HIV-2 Vpx in mammalian cells. (A)** HA-H11 degrades FLAG-Vpx. HEK293 cells were co-transfected with plasmids encoding FLAG-Vpx (50 ng) and HA-H11 (100 or 300 ng). Equal amounts of proteins for each sample were separated by SDS-PAGE and subjected western blot analysis using the indicated antibodies. The relative SAMHD1 expression level are shown as indicated. **(B)** HA-H11 expression does not effect on SAMHD1 expression. HEK293 cells were transfected with plasmid encoding HA-H11 (100 or 200 ng). Equal amounts of proteins for each sample were separated by SDS-PAGE and subjected western blot analysis using the indicated antibodies. The relative SAMHD1 expression levels are shown as indicated. **(C)** H11-dependent Vpx degradation is inhibited by MG132 treatment. HEK293 cells were co-transfected with plasmids encoding FLAG-Vpx (50 ng) and HA-H11 (200 ng), and then treated with or without 20 μM of MG132 treatment for 4 h. Equal amounts of proteins for each sample were separated by SDS-PAGE and subjected to western blot analysis using the indicated antibodies. **(D)** H11 degrades Vpx via its chaperone activity. HEK293 cells were co-transfected with plasmids encoding FLAG-Vpx and the indicated HA-H11 variants (WT, K113G, or K141E). Cell lysates were subjected to western blotting using the indicated antibodies. **(E)** FLAG-Vpx cycloheximide chase analysis in cells expressing HA-H11. HEK293 cells were co-transfected with plasmids encoding FLAG-Vpx and the indicated HA-H11 variant (WT, K113G, or K141E), and then treated with or without cycloheximide. Equal amounts of proteins for each sample were separated by SDS-PAGE and subjected to western blot analysis using theanti-FLAG or anti-Vinculin antibodies. Left panel shows quantitation of signal intensity.

Next, we asked whether H11 affects the stability of Vpx in cells. Transient transfection of FLAG-Vpx significantly decreased the level of endogenous SAMHD1 in HEK293 cells, as previously reported (**Figure 4A**, lanes 1 and 2) (Hrecka et al., 2011). Moreover, levels of FLAG-Vpx protein were reduced by co-transfection of HA-H11 protein in a dose dependent manner, resulting in restoration of SAMHD1 expression (**Figure 4A**). Overexpression of HA-H11 itself was no effect on endogenous SAMHD1 expression (**Figure 4B**). Degradation of Vpx protein by H11 was completely prevented by the proteasome inhibitor MG132 (**Figure 4C**). Next, we co-transfected HEK293 cells with FLAG-Vpx and WT H11, the kinase activity–deficient mutant K113G (Smith et al., 2000), or the chaperon-like activity–deficient mutant K141E (Shemetov and Gusev, 2011). Relative to vector control, WT H11 and the K113G mutant prominently reduced the level of Vpx, whereas the K141E mutant did not (**Figure 4D**). We then performed a cycloheximide (CHX) assay to determine the effect of H11 on the half-life of Vpx. In these experiments, HEK293 cells were co-transfected with FLAG-Vpx and HA-H11 WT or mutants, and then treated with CHX to inhibit translation. As shown in **Figure 4E**, the stability of FLAG-Vpx was significantly reduced by WT H11 and the K113G mutant, whereas expression of the K141E mutant had no effect. These results demonstrated that H11 promotes proteasome-dependent degradation of Vpx via its chaperone-like activity.

### H11 Suppresses HIV-2 Infection in Monocyte-Derived Macrophages

**FIGURE 5:**
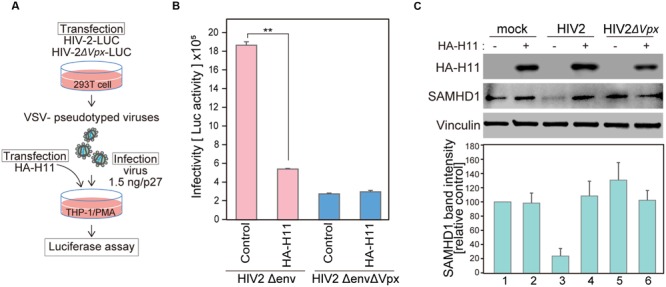
**H11 expression decreases single-round HIV-2 infection in MDMs. (A)** Schematic representation of the experimental system. **(B, C)** HEK 293T cells were co-transfected with pHIV-2Δenv-luc or pHIV-2ΔenvΔVpx–luc and with pVSV-G. Viral release was measured by quantitation of p27 antigen concentration in culture supernatants at 48 h post-transfection. THP-1 cells were differentiated with PMA for 24 h, and then transfected with plasmids encoding HA-H11 or empty vector (negative control). Twenty-four hours post-transfection, cells were infected with VSV- pseudotyped WT or ΔVpx viruses for 48 h. **(B)** Viral infectivity was detected by measuring luciferase activity in cell lysates. Data are means ± S.E.M. of three independent experiments. ^∗∗^*p* < 0.05, Student *t*-test. **(C)** Forty-eight hours after infection, cells were harvested and analyzed by western blotting using the indicated antibodies. Represent results from one of three independent experiments. Bar charts indicate amounts of SAMHD1, as determined by densitometric analysis of western blots. Data are means ± S.E.M. of three independent experiments.

Vpx degrades SAMHD1 in order to evade host intrinsic intracellular immunity and allow sustained HIV replication in myeloid cells (Goldstone et al., 2011; Hrecka et al., 2011; Laguette et al., 2011; Baldauf et al., 2012; Descours et al., 2012; Lahouassa et al., 2012). Therefore, we investigated whether H11-mediated Vpx degradation affects HIV-2 replication in monocyte-derived macrophages (MDMs). For this purpose, we used an HIV-2 clone harboring the luciferase gene (HIV-2-Δ*env*-LUC) and its *vpx*-deficient mutant (HIV-2Δ*env*Δ*vpx*-LUC) to produce chimeric viruses with the fusogenic envelope G glycoprotein of the VSV-G (**Figure 5A**). The infectivity of the generated viruses was tested using MDMs transfected with HA-H11 or vector alone. H11 overexpression suppressed infection of MDMs by WT HIV-2, but not Vpx-deficient HIV-2 (**Figure 5B**). SAMHD1 expression was significantly reduced in cells infected by WT HIV-2 (**Figure 5C**, lane 3), as reported in previous studies (Hrecka et al., 2011; Laguette et al., 2011). On the other hand, the level of SAMHD1 was restored in H11-transfected cells (**Figure 5C**, lane 4). These results suggested that H11 suppresses HIV-2 infection in MDMs via the up-regulation of SAMHD1-mediated virus restriction.

### H11 is Highly Expressed in Syncytiotrophoblast Cells

**FIGURE 6:**
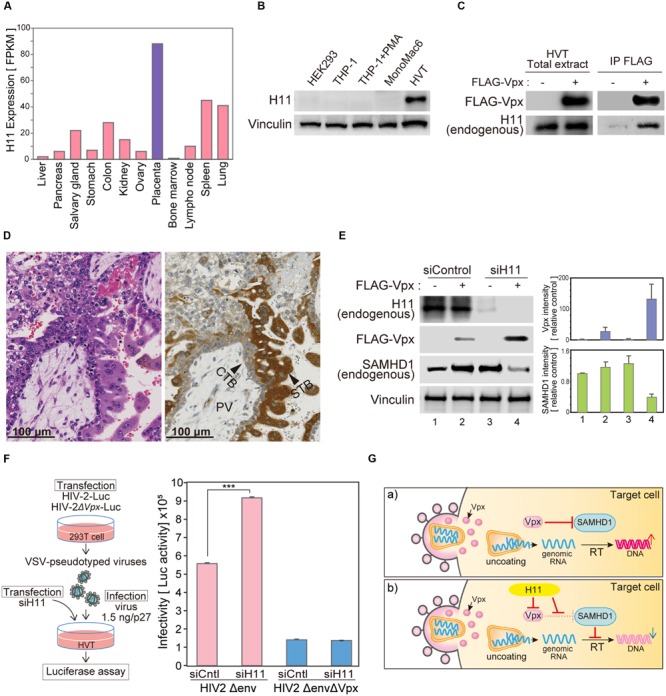
**H11 regulates HIV-2 infection in primary trophoblast cells. (A)** H11 mRNA levels in human organs and tissues. FPKM values for human H11 were obtained from the Human Protein Atlas Database (www.proteinatlas.org). **(B)** H11 protein expression in six cell lines (HEK293, THP-1 THP-1–derived macrophage, MonoMac 6, and HVT) were examined by western blot; vinculin was used as an internal control. **(C)** Immunoprecipitation of HIV-2 Vpx with H11 in HVT cells. HVT cells were transfected with FLAG-tagged HIV-2 Vpx, and cells were treated with 20 μM of MG132 from 6 h before harvest. Cells were harvested at 24 h after transfection, and lysates were immunoprecipitated with anti-FLAG. Input and immunoprecipitation samples were then probed with anti-H11 and anti-FLAG antibodies. **(D)** Immunohistochemistry (IHC) for detection of H11 in fourth week of human placental tissue. Brown color, IHC staining; blue color, hematoxylin counterstain. PV, placental villi; CTB, cytotrophoblast; STB, syncytiotrophoblast. Scale bars, 100 μm. **(E)** HVT cells were transfected with control or *H11*-targeted siRNA for 24 h, and then transfected with FLAG-Vpx. Twenty-four hours after transfection, cells were harvested and analyzed by western blotting using the indicated antibodies. Represent results from one of three independent experiments. Bar charts indicate the amounts of Vpx or SAMHD1, as determined by densitometric analysis of western blots. Data are means ± S.E.M. of three independent experiments. **(F)** HVT cells were transfected with control or *H11*-targeted siRNA for 24 h before infection with HIV-2. Twenty-four hours after infection, cells were harvested. Viral infectivity was detected by measuring luciferase activity in cell lysates. Data are means ± S.E.M. of three independent experiments. ^∗∗∗^*p* < 0.01, Student’s *t*-test. Left panel shows schematic representation of the experimental system. **(G)** Proposed model for H11-mediated regulation of HIV-2 in trophoblast cells. In the absence of H11, Vpx proteins are delivered into target cells by HIV-2 infection and degrade the host restriction factor SAMHD1, leading to productive infection. On the other hand, when H11 is present, it directly interacts with Vpx, leading to its degradation; consequently, SAMHD1 is liberated from Vpx and able to inhibit HIV-2 replication.

According to the Human Protein Atlas database, *H11* mRNA is significantly highly expressed in placenta (**Figure 6A**)^1^ (Uhlen et al., 2005, 2010, 2015; Berglund et al., 2008; Ponten et al., 2008). We analyzed H11 protein levels of in various cell lines. There was no obvious expression of H11 in the human monocyte/macrophage cell lines THP-1 and MonoMac6. By contrast, H11 was highly expressed in HVTs, a cell line derived from primary trophoblasts (**Figure 6B**). To test whether H11 can interact with Vpx in trophoblast cells, we performed an immunoprecipitation analysis using FLAG-Vpx transfected HVT cell extract. Endogenous H11 protein was co-precipitated with FLAG-Vpx (**Figure 6C**). These data prompted us to assess H11 expression in human placenta tissues. Immunohistochemistry (IHC) revealed that H11 was specifically expressed both in syncytiotrophoblast and cytotrophoblast cells, this was more prominently in syncytiotrophoblast, which form the border surface of placenta with the maternal circulation (**Figure 6D**).

### Targeted Depletion of H11 Restores Vpx Expression and HIV-2 Replication in a Primary Trophoblast Cell Line

Previous studies showed that the frequency of mother-to-infant HIV-2 transmission is very low (<2.5%) even in the absence of antiretroviral therapy during pregnancy (Padua et al., 2009; Burgard et al., 2010). Although the placenta acts as an effective barrier against HIV infection, according to the literature, the underlying molecular mechanisms have not been well characterized. We hypothesized that H11 is functionally involved in the defense against HIV infection at the placenta. To test this idea, we used siRNA targeting *H11* to inhibit endogenous H11 expression in HVT cells. In transduced HVTs, exogenous expression of FLAG-Vpx was relatively low, but increased dramatically following *H11* siRNA transfection (**Figure 6E**). Moreover the level of endogenous SAMHD1 was reduced in concert with an increase of Vpx expression (**Figure 6E**). To further explore the relevance of this phenomena, we asked whether knockdown of *H11* would affect HIV-2 replication. As a replication marker, we measured luciferase activity in cells infected with HIV-2 reporter virus. The luciferase activity of HIV-2 Luc was elevated in *H11*-suppressed HVT cells, whereas that of the Vpx-null (HIV-2ΔVpx) virus was not significantly affected (**Figure 6F**). These results further support the idea that HIV-2 replication in human trophoblast cells is negatively regulated by H11-mediated degradation and functional disruption of Vpx.

## Discussion

Identification of new virus–host cell interactions is a key challenge in clarifying the nature of viral infection and pathogenesis. An understanding of the molecular mechanisms underlying the virus–host interaction will facilitate the development of new therapeutic strategies against viral infect ions. Thus, establishment of assay system for comprehensive analysis of virus–host protein interaction may lead to new advances in virus research.

In this study, we developed a new assay system that combines wheat germ CFPS and the AlphaScreen system. This method enabled us to comprehensively screen for any kind of robust virus–host protein interaction. As an illustration, we utilized our system to identify a host factor that interacts with HIV-2 Vpx. We found that H11 interacts with HIV-2 Vpx and promotes its degradation via a proteasome-dependent pathway. In addition, we demonstrated that H11 is highly expressed in human trophoblasts and may prevent maternal-to-fetal transmission of HIV-2 during pregnancy. Thus, our proteomic approach is an efficient and powerful tool for revealing biologically or pathologically relevant molecular events in virus infection.

We exploited the AlphaScreen system to systematically measure virus–host protein interactions. One major advantage of the AlphaScreen technology is that it is a homogeneous (no-wash) assay and can be applied to crude samples such as tissue homogenates, cell lysates, cell culture supernatants, and cell extracts. AlphaScreen beads can only recognize the objective proteins with a specific epitope tag, permitting us to use non-purified proteins synthesized in a wheat cell-free system. This advantage saves considerable time and labor on protein extraction and purification.

For reliable and efficient screening of protein–protein interactions, it is necessary to use proteins with proper tertiary structures and biological functions. Wheat germ CFPS is a eukaryotic translation system that synthesizes proteins that are properly folded and biologically active, as in living mammalian cells. Another desirable property of wheat germ CFPS is its suitability for viral protein production. Viral proteins are usually insoluble and form aggregates in inclusion bodies in living cells. Moreover, viral proteins are generally cytotoxic and induce cell death upon expression. The wheat germ cell-free system is able to produce proteins that are relatively insoluble in other systems because it can tolerate alterations in buffer components, including adjustments to salt concentration and/or addition of various detergents. These advantages underscore the suitability and availability of the wheat germ CFPS for the generation of viral proteins that can be used for comprehensive protein–protein interaction assays.

We introduced the wheat germ CFPS to provide a set of recombinant proteins from a cDNA library of human protein kinases. Our system can produce proteins from linear cDNA templates generated by PCR (Sawasaki et al., 2007; Matsuoka et al., 2010; Matsunaga et al., 2015). Moreover, when a biotin ligation sequence is inserted into cDNAs by PCR, biotinylated proteins can be readily synthesized in the presence of biotin and biotin ligase. The biotinylated proteins are readily discernible by streptavidin, which has high affinity for biotin, and clearly recognized in a homogenous condition such as AlphaScreen assay system (Sawasaki et al., 2008; Matsuoka et al., 2010). Based on these features and the aforementioned advantages, we can perform high-throughput assays under optimal experimental conditions using selective cDNA libraries, e.g., the human protein kinase library used in this study.

HIV-2 infection occurs mainly in West Africa, but its prevalence is increasing in Europe, India, and the United States. Compared with HIV-1, HIV-2 exhibits a longer asymptomatic phase, slower progression to terminal immunosuppression, and lower efficiency of both transmission and replication (∼30-fold lower than HIV-1; Popper et al., 1999; MacNeil et al., 2007; Gottlieb et al., 2008; Gueudin et al., 2008; Nyamweya et al., 2013; Menendez-Arias and Alvarez, 2014). These observations suggest the existence of different modes of host–virus interaction in HIV-2 infection. HIV-2 encodes an accessory protein, Vpx, degrades the cellular restriction factor SAMHD1, which strongly inhibit viral replication in non-dividing cells (Berger et al., 2011; Hrecka et al., 2011; Laguette et al., 2011). Moreover, Vpx enhances nuclear import of the pre-integration complex following infection (Shingai et al., 2015). Vpx is thus essential to maintenance of productive HIV-2 infection in non-dividing cells. However, it remains unclear how Vpx function is regulated in terms of virus–host interaction. Therefore, we focused on Vpx as a target of our proteomic analysis to elucidate host molecular mechanisms involved in the functions and regulation of Vpx in HIV-infected cells.

Viruses can pass through the placental barrier from maternal to fetal blood. A pregnant woman infected with HIV can transmit the virus to her fetus at any time during pregnancy. Previous reports showed that trophoblastic cells of human placenta tissue express CD4 and/or chemokine receptors, such as CCR-5 and CXCR-4, and susceptible to HIV infection (Amirhessami-Aghili and Spector, 1991; Ishii et al., 2000), and both HIV-1 and -2 have been detected *in situ* in placental syncytiotrophoblasts of HIV-positive pregnant women (Lewis et al., 1990; Backe et al., 1992). However, placental HIV-1 and -2 infection during the neonatal period seems to be rare, although the risk of infection becomes much higher during labor and vaginal delivery (Leroy et al., 2002; Padua et al., 2009; Burgard et al., 2010). These results suggest the presence of novel restriction factor(s) in trophoblasts that are capable of suppressing maternal-to-fetal transmission of HIV. In this study, we identified H11 as a putative restriction factor that may suppress HIV-2 replication in THP-1 and trophoblastic cells (**Figure 6G**). Our proteomic analysis revealed that H11 can bind directly to Vpx and promote its proteasomal degradation. Consequently, the host restriction factor SAMHD1 is liberated from Vpx and can suppress HIV-2 replication. H11 is not expressed in immune cells such as MDMs and T cells, but is highly expressed in human placental trophoblastic cells, to a greater extent in the outer syncytiotrophoblast rather than in the inner cytotrophoblast. Previous reports suggested that the antiviral activity of SAMHD1 is limited to non-dividing cells such as terminally differentiated myeloid cells and quiescent CD4+ T lymphocytes. Because syncytiotrophoblasts are generally fully differentiated and non-dividing cells, H11 may give these cells the ability to suppress HIV-2 transmission. Further careful analysis with human samples is necessary to delineate the biological function of H11 in HIV infection.

In summary, we developed a novel protein–protein interaction assay system that combines the wheat germ CFPS system with AlphaScreen technology. Our assay system provides rapid and reliable experimental format for uncovering novel virus–host protein interactions. Further evaluation using different viral species and different sets of human cDNA libraries could validate the utility and benefit of this system for future virological research.

## Author Contributions

AK designed and performed the experiments and wrote the manuscript. KM accumulated data and performed analysis. SM performed *in vitro* assays and analyzed data. YM analyzed the computational binding model. IK performed and analyzed immunohistochemistry. HK provided reagents and advice on the project. SH provided key materials. KM and TS generated initial observations of this project. AR designed and supervised the research, analyzed the data, and wrote the manuscript. All authors participated in drafting, revising and final approval of the manuscript, and agree to be accountable for all aspects of the work.

## Conflict of Interest Statement

The authors declare that the research was conducted in the absence of any commercial or financial relationships that could be construed as a potential conflict of interest.

## Footnotes

1 www.proteinatlas.org
